# Classification of Plant-Based Drinks Based on Volatile Compounds

**DOI:** 10.3390/foods13244086

**Published:** 2024-12-17

**Authors:** Zsigmond Papp, Laura Gabriela Nemeth, Sandrine Nzetchouang Siyapndjeu, Anita Bufa, Tamás Marosvölgyi, Zoltán Gyöngyi

**Affiliations:** 1 Department of Public Health Medicine, Medical School, University of Pécs, Szigeti út 12, 7624 Pécs, Hungary; papp.zsigmond@pte.hu (Z.P.); laura.nemeth@pte.hu (L.G.N.); sandrine.nzetchouangsiyapndjeu@aphp.fr (S.N.S.); 2 Faculty of Health Sciences, University of Pécs, Vörösmarty u. 4, 7621 Pécs, Hungary; 3 Recherchenité de Recherche Clinique, GHU Paris Centre, Université Paris Cité, 89 rue d’Assas, 75006 Paris, France; 4 Institute of Bioanalysis, Medical School, University of Pécs, Szigeti út 12, 7624 Pécs, Hungary; anita.bufa@aok.pte.hu (A.B.); marosvolgyi.tamas@pte.hu (T.M.)

**Keywords:** plant-based drinks, classification, gas chromatography ion mobility spectrometry, GC-IMS, electronic nose, e-nose

## Abstract

The increasing popularity of plant-based drinks has led to an expanded consumer market. However, available quality control technologies for plant-based drinks are time-consuming and expensive. Two alternative quality control methods, gas chromatography with ion mobility spectrometry (GC-IMS) and an electronic nose, were used to assess 111 plant-based drink samples. Principal component analysis (PCA) and linear discriminant analysis (LDA) were used to compare 58 volatile organic compound areas of GC-IMS gallery plots and 63 peptide sensors of the electronic nose. PCA results showed that GC-IMS was only able to completely separate one sample, whereas the electronic nose was able to completely separate seven samples. LDA application to GC-IMS analyses resulted in classification accuracies ranging from 15.4% to 100%, whereas application to electronic nose analyses resulted in accuracies ranging from 96.2% to 100%. Both methods were useful for classification, but each had drawbacks, and the electronic nose performed slightly better than GC-IMS. This study represents one of the first studies comparing GC-IMS and an electronic nose for the analysis of plant-based drinks. Further research is necessary to improve these methods and establish a rapid, cost-effective food quality control system based on volatile organic compounds.

## 1. Introduction

Plant-based drinks, which have a consistency similar to milk, are not officially classified as a novel food. These non-dairy beverages represent appealing alternatives to traditional dairy products, particularly among consumers who keep a vegetarian or vegan diet and those who are lactose intolerant or allergic to dairy proteins [1,2]. Plant-based drinks also have a more favorable environmental impact than dairy milk production, including reduced greenhouse gas emissions and lower water use. These attributes have led to the increasing popularity of plant-based drinks, expanding their market share and leading to the introduction of plant-based drinks in a wide range of flavors [2,3]. Most plant-based drinks are derived from soy and almond products, but plant-based drinks have been developed [4] from cereals, legumes, nuts, seeds, and pseudo-cereals [5], with diverse nutritional qualities and variations in macro- and micronutrients [4,6,7].

The nutritional properties of plant-based drinks are determined by three primary characteristics: (1) the raw source materials [8]; (2) the formation, which describes the addition of artificially constructed or isolated oil bodies to simulate the fat globules found in dairy milk; and (3) the formulation [9], which refers to the other functional ingredients, such as coloring and flavoring agents, preservatives, thickeners, stabilizers, minerals, and vitamins. All of these components determine the nutritional contents of plant-based drinks [9], and their proportions may vary across brands.

Plant-based drinks intended for human consumption are required to meet food quality control standards. However, traditional analytical methods used for evaluating food quality and safety, such as gas chromatography (GC), mass spectrometry (MS), and atomic absorption spectroscopy, often require pre-concentrated samples, are expensive and time-consuming, and necessitate a well-equipped laboratory staffed by skilled personnel [10,11]. A new, non-destructive analytical method involves the coupling of spectroscopy with near-infrared and hyperspectral imaging techniques (NIR spectroscopy), but this approach remains limited by technical challenges [12].

Intelligent sensory instruments, such as electronic tongues or noses, represent potentially cost-effective options for gathering information on the chemical composition of a sample. Thorough chemical analyses can be conducted rapidly by assessing small sample volumes using these instruments. Such analyses could be used to identify all ingredients in a plant-based drink, enabling the rapid differentiation of drinks according to ingredients and facilitating shelf-life estimates, identification of adulterations, and assessments of authenticity [13,14].

Another option for assessing food quality is ion mobility spectrometry (IMS), in which different compounds within a sample are ionized, and their migration is evaluated [15]. Because mobility depends on the mass, shape, and size of ionized compounds, each sample will generate a unique chemical or molecular fingerprint that can be used to compare samples for the purposes of identification, authentication, or fraud detection [15].

We intended to compare an unsupervised and a supervised method to reduce the number of dimensions. The two most popular methods are principal component analysis (PCA) and linear discriminant analysis (LDA) [16].

The present study assessed two promising, rapid, efficient, and inexpensive analytical methods, an electronic nose and GC-IMS, and compared their abilities to differentiate among and classify multiple plant-based drinks containing different source materials produced by different brands.

## 2. Materials and Methods

### 2.1. Plant-Based Drink Sample Selection

We purchased 111 plant-based drinks from local supermarkets (ALDI, Auchan, DM, EcoFamily, Penny Market, SPAR, and TESCO in Pécs, Hungary). All samples were stored at −80 °C to maintain their chemical properties until sensory analysis. The investigated samples were produced by thirteen companies: Adez, Alnatura, Alpro, DMbio, Happy, Isola, Joya, Koko, My Bio, Natur Aktiv, Plant Pro, Riso Scotti, and The Bridge. The main ingredients of each sample are listed in Table 1. All plant-based drinks can be classified into one of seven categories based on their primary source: almond, cashew, coconut, oat, rice, soy, or spelt.

All samples were categorized as either organic or conventional based on their labeling. A sample was considered an organic product if the product packaging stated it was generated from plants grown using organic farming approaches, which forbid the use of artificial synthetic pesticides, fertilizers, or genetically engineered products [17]. All other samples were considered conventional products. Samples labeled as intended for commercial use (by baristas) were grouped into a specific “barista” subgroup. Samples were also classified according to roasting, which is part of the manufacturing process. Additional information, such as the addition of sweeteners or calcium, the use of ultra-high temperature (UHT) pasteurization, and the presence or absence of gluten, were used to categorize the samples. All essential categories are presented in Table 1.

### 2.2. Sample Preparation

#### 2.2.1. Sample Preparation for GC-IMS

All samples were stored at −80 °C until analysis. Prior to analysis, samples were thawed at room temperature until no solid phase was observable, after which they were carefully shaken. The container was opened for a short time, and the headspace volume in the original tubes was minimized. A 1 mL volume of each sample was removed from the original container and placed in a 20 mL plastic screw-capped vial. Based on the findings of a prior study [18], 500 mg of sodium chloride was added to each sample to help analytes move to the headspace.

#### 2.2.2. Sample Preparation for the NeOse Pro Electronic Nose

The ‘Happy’ almond-based sample, which has a neutral taste and smell and contains only 1% almond product, was used as a control for every electronic nose measurement. All samples, including the control sample, were stored at −80 °C until analysis. Prior to analysis, all samples were thawed at 4 °C. After thawing, 1 mL of each sample was placed into each of seven labeled vials. Sealed vials were incubated at 95 °C for 40 min and then allowed to cool at room temperature (25 °C) for 20 min.

### 2.3. Analysis

#### 2.3.1. GC-IMS Analysis

The protocols were optimized prior to GC-IMS and electronic nose measurements to achieve the highest volatile concentration and high instrument sensitivity. The original manufacturer’s test applied 40 °C for the GC column and 45 °C for IMS with 20 min of incubation of samples at 60 °C. During the optimization, we tested 40, 60, and 95 °C incubation temperatures and changed the IMS temperature to 70 °C because we expected higher sensitivity. Based on the test results, 40 °C for GC, 70 °C for IMS, and 95 °C incubation temperatures were chosen. Samples were incubated for 20 min at 95 °C. During the first 10 min, caps were opened slightly to prevent an explosion, and caps were closed completely for the last 10 min. No controls were included in GC-IMS analyses.

The BreathSpec GC-IMS device (Gesellschaft für Analytische Sensorsysteme, G.A.S., GmbH, Dortmund, Germany) consists of a core component (G.A.S, Dortmund, Germany) equipped with a wide-bore GC column (MXT-WAX 30 m × 0.53 mm, RESTEK, Bellefonte, PA, USA).

A heated (95 °C) 5 mL plastic syringe with a 51 mm needle was used to collect 1 mL of headspace from each sample, and 200 µL of the collected headspace was injected into a heated (95 °C) splitless injector. After injection, analytes were passed into the GC column for the first separation. The eluate was transferred to another column for a second separation using the IMS, which was equipped with a tritium ionizing radioactive source (5000 eV) and a 9.8 cm long drift tube. The drift gas had a flow rate of 150 mL/min and a pressure of 0.712 kPa. The carrier gas flow rates are shown in Table 2.

VOCal software Version 0.1,3 (Gesellschaft für Analytische Sensorsysteme GmbH, G.A.S.; Dortmund, Germany) was used to select 58 areas from among the measured volatile compounds and highlight their respective signals in the chromatogram. Area 66, the reference signal registered by the GC-IMS device, was used to normalize the intensities of all 58 areas in every sample, and the resulting ratios were used in further analyses.

#### 2.3.2. Analysis Using the NeOse Pro Electronic Nose System

All samples, including the control sample, underwent comprehensive analysis using the NeOse Pro electronic nose system (Aryballe Technologies, Grenoble, France), which consists of an optoelectronic sensor array with 63 non-specific peptides printed on a gold layer [19]. A dynamic measurement was conducted with the following parameters: a pump flow rate of 50 mL/min, 25 frames per second, and a core temperature of 32 °C. The time required to measure one sample was approximately 2 min, ensuring a thorough and high-quality analysis.

**Figure 1:**
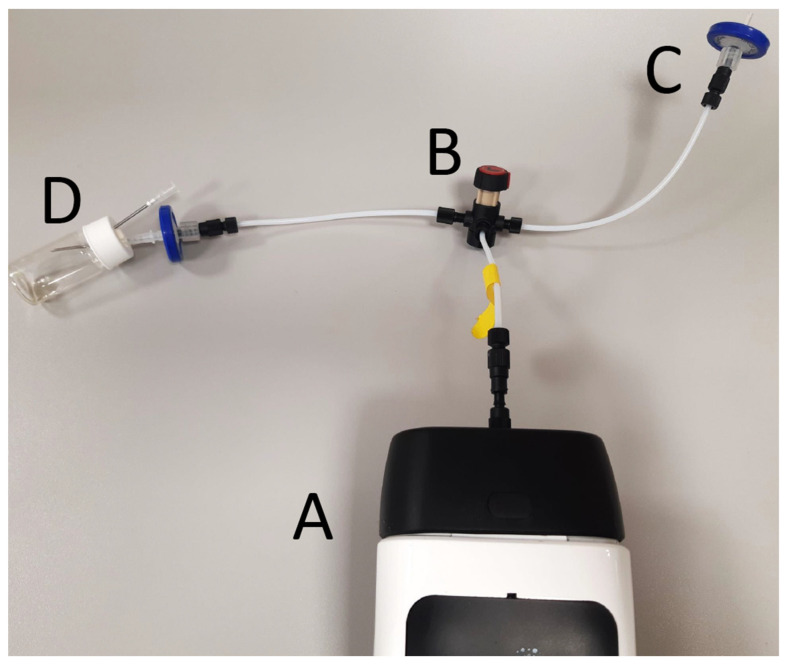
Operation setting of the electronic nose. Tubing is connected to the electronic nose (A) with a switcher (B). Membrane filters (C) provide filtered air. The headspace of a 20 mL glass vial holding 1 mL sample is taken through a needle; an extra needle provides air to avoid creating a vacuum (D).

A total of six aliquots of every sample were measured. Control samples were measured once at the beginning, twice during each session, and once at the end, and the control sample measurements were used as reference points to calibrate the results. The first two measurement sessions for each sample were necessary to saturate the polytetrafluoroethylene membrane (32 mm diameter, 0.45 µm RephiQuick Syringe Filter; RephiLe Bioscience Ltd., Zhejiang, Shanghai, China) protecting the sensors; therefore, the first two measurements for each sample were discarded. The operation setting is seen in Figure 1.

### 2.4. Statistics

The mean control sample value was subtracted from each sample value to correct for any drift in the electronic nose. The absolute value of the lowest negative value was added for every value in the corrected results to eliminate the negative values in the dataset.

PCA and LDA were conducted on GC-IMS and electronic nose data to classify each sample. For the PCA scatterplot, we used ClustVis, a web tool for visualizing the clustering of multivariate data. A confidence level of 0.95 defines the ellipses [20]. Fisher’s coefficient and the Mahalanobis distance with stepwise analysis were utilized for the LDA, using IBM SPSS Statistics for Windows, Version 28 (IBM, Armonk, NY, USA).

Samples were compared across brands (Alpro: almond, cashew, rice, and sugar-free soy; DMbio: almond, coconut, oat, rice, soy, and spelt), type (barista: almond, coconut, and oat), and plant source (almond: roasted, barista, conventional and organic; coconut: Adez, Joya, Koko, Naturaktiv, and Happy; conventional rice: Alpro traditional, Alpro sweetened, PlantPro, and Happy; organic rice: Auchan, Isola, Riso Scotti, DMbio, The Bridge, and MyBio).

The data presented in the figures, illustrating gallery plots or PCA/LDA results, did not undergo standardization or normalization across samples.

## 3. Results

### 3.1. Comparison of PCA Results

**Figure 2:**
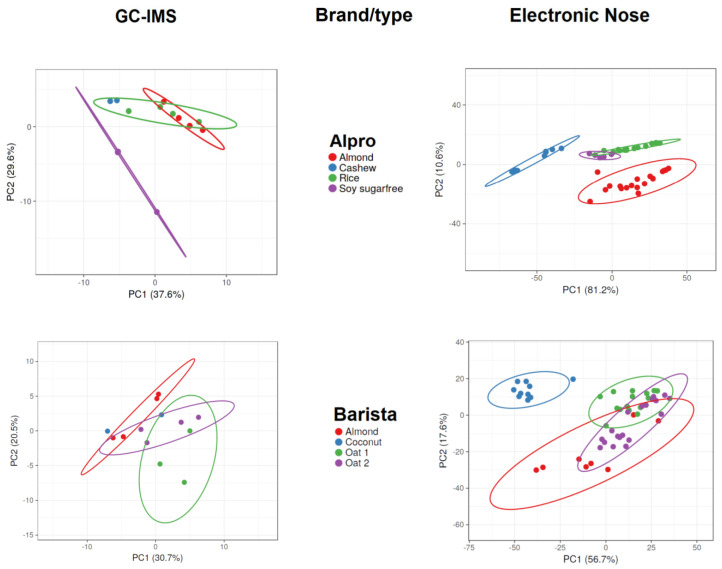

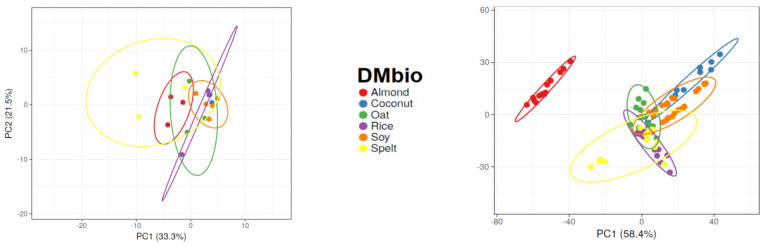
Principal component analysis results of gas chromatography with ion mobility spectroscopy (GC-IMS) and electronic nose measurements of plant-based drinks according to brand and type.

**Figure 3:**
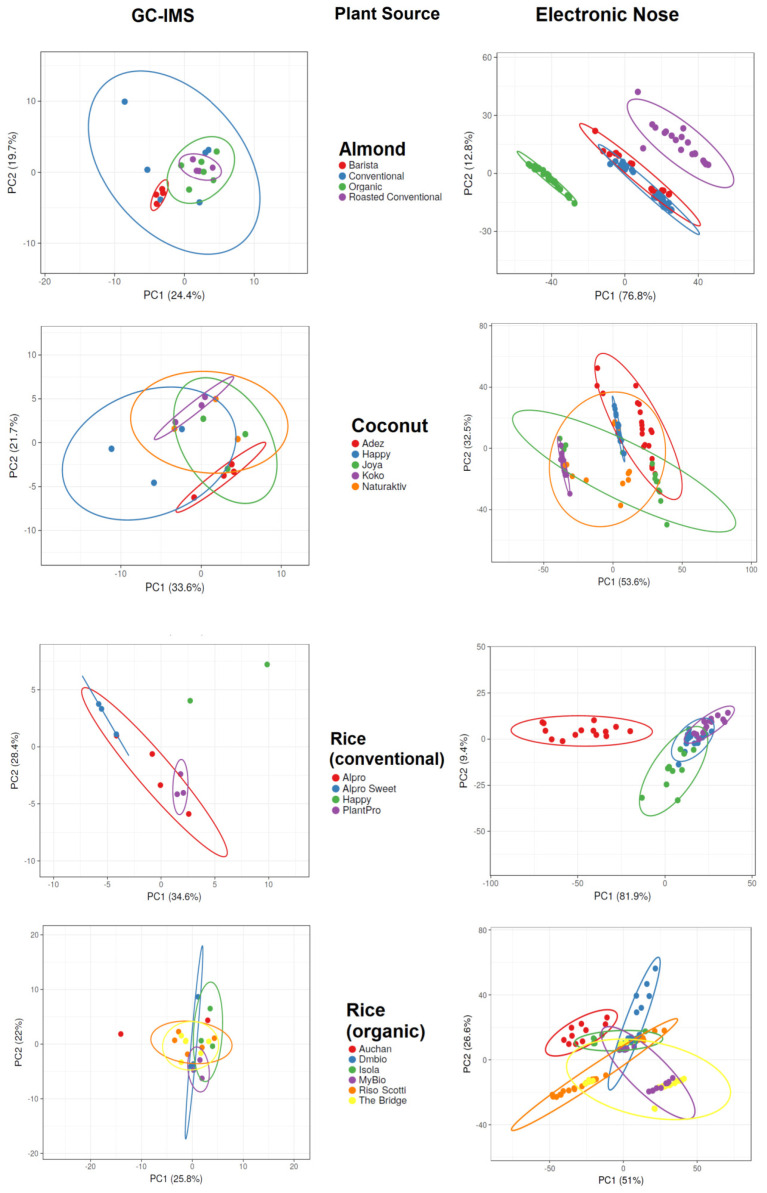
Principal component analysis results of gas chromatography with ion mobility spectroscopy (GC-IMS) and electronic nose measurements of plant-based drinks according to plant sources.

Figure 2 and Figure 3 display the PCA results for the data acquired with GC-IMS and the electronic nose. The PCA results show more overlap and lower separation between groups when applied to GC-IMS data than when applied to electronic nose data. When assessing Alpro samples, the GC-IMS approach was able to completely separate sugar-free soy samples from other Alpro samples, and this approach was also able to separate cashew-based samples from almond-based samples. However, overlap occurred between almond-based and rice-based samples and between cashew-based and rice-based samples. The electronic nose approach was able to completely separate almond-based and cashew-based samples from other Alpro samples, whereas overlap occurred between rice-based and sugar-free soy Alpro samples.

When assessing barista samples, the GC-IMS approach was able to separate almond-based samples from one of two oat-based samples, but no complete separation was possible when comparing all barista samples using this approach. By contrast, the electronic nose approach was able to completely separate coconut-based samples from all other barista samples, but overlaps occurred between almond-based samples and the two oat-based samples.

The GC-IMS approach was not able to completely separate any of the DMbio samples, although some separation was observed between almond-based and rice-based samples. However, overlaps occurred across all sample types assessed using GC-IMS. The electronic nose approach was able to separate almond-based samples from every other DMbio sample, and separation could be observed between rice-based and coconut-based samples; however, these samples overlapped with every other sample type except almond-based samples.

GC-IMS of almond-based samples was able to separate barista samples from roasted conventional samples, but no complete separation was possible when all almond-based samples were analyzed together. The electronic nose analysis of almond-based samples was able to separate organic and roasted conventional samples from all other almond-based samples, but an overlap occurred between barista and conventional samples. GC-IMS analysis of coconut-based samples was unable to completely separate any sample when all samples were analyzed, but separation was observed between the Adez and Koko samples. The electronic nose was also unable to completely separate any coconut-based samples when all samples were analyzed, but separation was observed between the Koko and Happy samples and between the Koko and Adez samples. GC-IMS analysis of conventional rice samples was able to completely separate the Happy sample from all other samples, and separation was observed between the sweetened Alpro and PlantPro samples. The electronic nose was also able to completely separate Alpro conventional rice samples from all other samples, but overlap was observed among the sweetened Alpro, Happy, and PlantPro samples. GC-IMS analysis was able to separate Auchan organic rice samples from MyBio organic rice samples, but incomplete separation was observed when all organic rice samples were assessed. The electronic nose was able to separate Auchan organic rice samples from Riso Scotti and DMbio organic rice samples, but no complete separation was visible when all organic rice samples were assessed.

Carob flour, vitamin E, sunflower–lecithin, vitamin E, calcium carbonate, and sugar were among the ingredients in overlapping products measured by GC-IMS but never the ingredients of overlapping groups measured by electronic nose. This is indirect evidence, but it is possible that the electronic nose is more sensitive to these products or impurities belonging to these products than the GC-IMS. It is also possible that other sources were responsible for the better classification (Table 3).

### 3.2. Comparison of LDA Results

**Figure 4:**
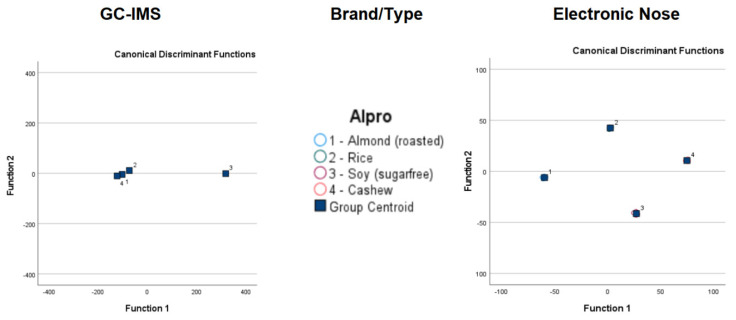

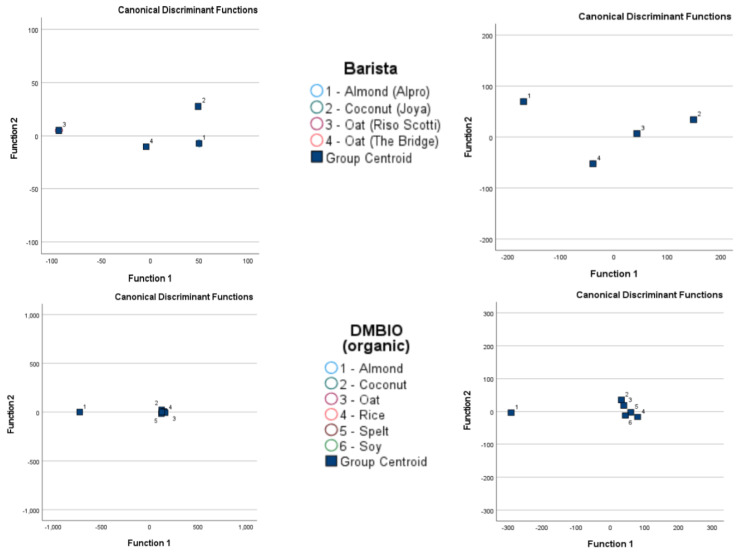
Linear discrimination analysis classification of gas chromatography with ion mobility spectroscopy (GC-IMS) and electronic nose analysis of plant-based drinks according to brand and type.

**Figure 5:**
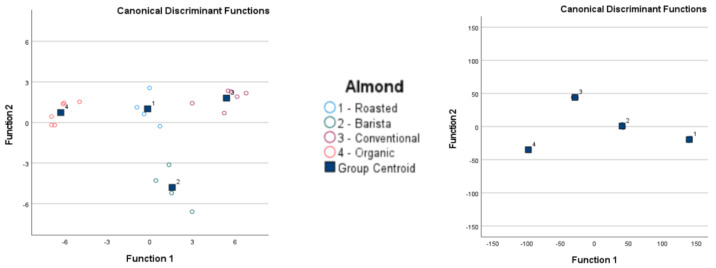

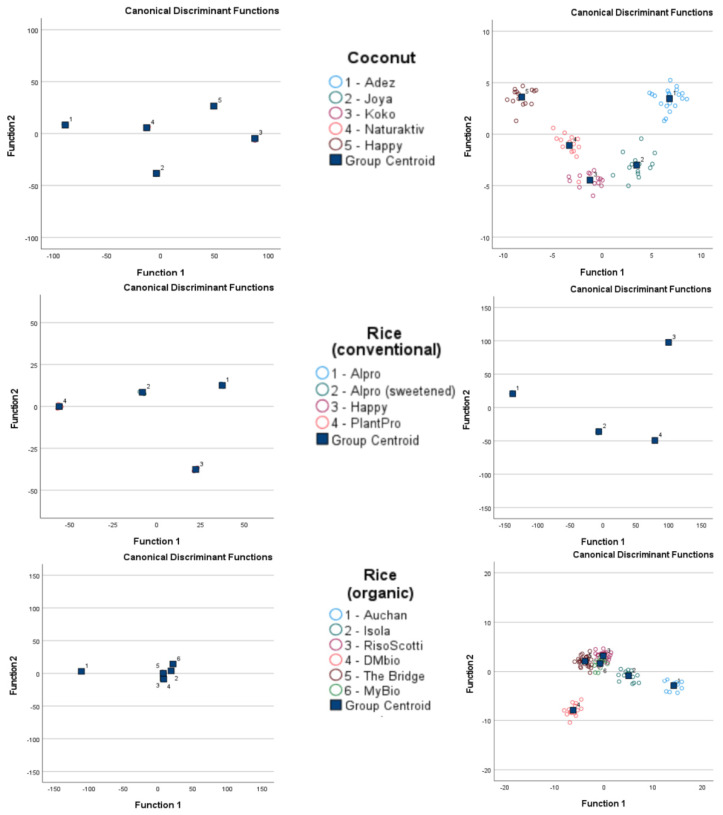
Linear discrimination analysis classification of gas chromatography with ion mobility spectroscopy (GC-IMS) and electronic nose analysis of plant-based drinks according to plant source.

Table 4 compares the LDA results before and after cross-validation. Figure 4 and Figure 5 show visual comparisons of the LDA results. Detailed classification results are in the Supplementary Materials (Tables S1–S14).

After cross-validation, the LDA classification results for GC-IMS were less accurate than those for the electronic nose. Although the lowest accuracy for GC-IMS classification was 15.4% for Alpro samples, all other accuracy values were 89.5% or higher. However, higher accuracy values were obtained overall for electronic nose results, with the lowest observed accuracy at 96.2%.

## 4. Discussion

Both GC-IMS and electronic nose approaches are able to precisely and thoroughly detect volatile organic compounds [21]. Volatile compounds have been successfully used to identify the primary sources of various plant-based drinks, including rice [22], oat [23], almond [24], coconut [25], soy [26], cashew [27], and cereals [28]. Volatile compounds have also been successfully used to identify additional ingredients in plant-based drinks, such as sunflower oil [29], pea protein [30], algae [31], grape juice [32], and agave syrup [33]. For example, these approaches have been used to successfully differentiate among room-temperature yogurt, non-fermented plant-based drinks, and fermented plant-based drinks made from walnut and purple rice, with complete separation visible when PCA was applied to electronic nose results and GC-IMS gallery plots [34]. Our comprehensive study demonstrates that the presence of volatile organic compounds identified using GC-IMS or electronic nose approaches heavily influences the PCA-based differentiation of plant-based drinks.

Few studies have explored the analysis of plant-based drinks using GC-IMS or electronic nose approaches, although one study was able to differentiate among four different samples of water-boiled salted duck, with PCA of both electronic nose and GC-IMS results leading to complete separation of all four samples [35]. Another study used GC-IMS and electronic nose approaches to explore changes in volatile compounds after the fermentation of traditional Chinese shrimp pastes, and PCA results showed both separation and overlap among various samples [36]. A study using GC-IMS and electronic nose approaches to explore changes in aroma characteristics across grass carp mince samples subjected to different washing processes found that PCA could separate samples completely [37]. These studies demonstrate that both of these approaches may be able to provide valuable data for classification purposes. The present study is among the first studies to compare the use of GC-IMS and electronic nose approaches for differentiating among various plant-based drink samples. In our study, most almond samples were classified correctly, with 95–100% accuracy. The incorrect classification may result from using a low percentage of almonds, which ranged between 1% and 7% in our samples. The other reason for incorrect classification may derive from mixed products. For instance, coconut and rice or coconut and soybean are used together. Producers may use different protocols and ingredients, but there are common ingredients and additives, like sunflower oil, gellan gum, and vitamin B2. Quality control measures are needed to ensure the quality and safety of plant-based drinks intended for human consumption [10]. One step in the quality control process applied to food is identifying the origins of the food product [14], which may be conducted using GC-IMS [10,15,38] and electronic nose [14,39] approaches. However, our results indicate that in the absence of GC-IMS fingerprint references, GC-IMS was unable to clearly differentiate among various plant-based drinks. By contrast, the electronic nose approach was able to differentiate among different plant-based drinks more efficiently than GC-IMS. In the present study, the electronic nose approach was both more accurate and quicker than the GC-IMS approach, able to analyze a single sample in 2 min compared with the 25 min required for GC-IMS. Based on the characteristics of GC-IMS and the electronic nose tested, we suppose that GC-IMS is more sensitive to apolar molecules while the electronic nose is more sensitive to polar molecules. The headspace of plant-based drinks was likely more abundant in polar molecules in our study. The classifiers were different at the electronic nose and GC-IMS. The GC-IMS provided concentration data of volatile molecules, while the sensors, binding various molecules, were the variables at the electronic nose. Our results indicate that the GC-IMS and the electronic nose approaches should be improved to replace the complex systems currently used to assess food quality control, such as GC-MS and liquid chromatography–MS. However, both GC-IMS and the electronic nose approaches could be integrated into more sophisticated systems [40,41] or be used to conduct preliminary tests when the packaging has no label, is suspected of being mislabeled, or if fraud or adulterations are suspected.

If improvements in these two approaches lead to high reproducibility and standardization of plant-based drink analyses, these approaches could represent options for rapid quality monitoring during product manufacture, enabling producers to verify and maintain quality standards for plant-based drinks. If samples or batches fail to be classified correctly, they may be flagged for more thorough examinations, helping producers eliminate potential problems during production, including spoilage, contamination, and the lack of or addition of excess ingredients. Authorities could use similar systems to evaluate suspected cases of adulterations or mislabeling.

## 5. Conclusions

Plant-based drinks are increasingly popular, and drinks are being produced from multiple plant-based sources. Quality control is essential for plant-based drinks intended for human consumption, and the development of rapid, reliable, and cost-effective methods could help producers and authorities detect mislabeling and adulterations before they cause harm. GC-IMS devices and electronic noses are portable devices, which makes them desirable for such analyses. In our study, we assessed the accuracy of GC-IMS and electronic nose analyses for differentiating between plant-based drinks according to source plant or brand. The electronic nose was more accurate and had a more favorable time–cost ratio than the GC-IMS approach. Further research is needed to determine the accuracy and reproducibility of each approach. Better results could lead to standardization and the establishment of a fast and efficient monitoring system.

## Figures and Tables

**Table 1:** Description of analyzed samples. UHT, ultra-high temperature pasteurization. The samples are numbered in the order of purchase (last four columns).

Source	Type	Brand	Additional Information	Main Labeled Ingredients	Sample Number			
Almond	Conventional	Adez		almond (2%), sunflower lecithin, gellan gum, steviol glycosides, vitamins (B_12_, D)	10	29	61	
Almond	Conventional	Plant Pro	UHT, with added calcium and minerals	almond (2.75%), sugar, sunflower oil, calcium carbonate, salt, sunflower lecithin, vitamins (A, D, E)	73	111	113	
Almond	Conventional	Happy		almond (1%), calcium carbonate, guar gum, gellan gum, lecithins, salt	149	181	199	
Almond (roasted)	Conventional	Alpro		almond (2%), salt, sugar, sunflower lecithin, carob flour, gellan gum, vitamins (B_2_, B_12_, D, E)	89	91	106	124
Almond	Conventional (Barista)	Joya		almond (2.5%), gellan gum, maltodextrin, lecithins, salt	144	178	204	
Almond	Conventional (Barista)	Alpro		almond (2.5%), sugar, fructose, calcium carbonate, guar gum, gellan gum, salt	22	46	65	
Almond	Organic	DMbio	UHT; natural	almond (7%), salt	68	76	114	
Almond	Organic	The Bridge	gluten-free	Italian almond paste (3%), cane sugar, carob flour	56	74	103	
Almond	Organic	Happy		almond (1%), calcium carbonate, guar gum, gellan gum, lecithins, salt	181	149	199	
Cashew	Conventional	Alpro		cashew (3.1%), sea salt, carob flour, gellan gum, sunflower lecithin, vitamins (B_2_, B_12_, D_2_, E)	11	42	52	
Coconut	Conventional	Adez		coconut extract (4.6%), rice (3.8%), sunflower lecithin, gellan gum, guar gum, vitamins (B_12_, D)	38	41	53	58
Coconut	Conventional	Joya	UHT, with added calcium	coconut milk (coconut cream, water) (5.3%), rice (3.8%), gellan gum, guar gum, lecithins, salt, vitamins (B_12_, D_2_)	67	71	79	85
Coconut	Conventional	Koko	dairy-free; original recipe	coconut milk (8.4%), grape juice concentrate, fatty acid–sucrose esters, salt, carotene, vitamins (B_12_, D_2_)	86	102	121	
Coconut	Conventional	Happy	new recipe	coconut (2%), rice (3.8%), gellan gum, guar gum, lecithins, salt	136	161	172	
Coconut	Conventional (Barista)	Joya	gluten-free	coconut milk (coconut cream, water) (9%), soybean (2.3%)	151	214		
Coconut	Organic	Natur Aktiv		coconut meat (12%), agave syrup, guar gum, salt	70	95	123	
Coconut	Organic	DMbio	UHT	coconut (8%), sea salt	14	27	50	59
Oat	Organic (Barista)	Riso Scotti		oat (16%), sunflower oil, pea protein, salt	128	185	192	
Oat	Organic (Barista)	The Bridge		oat (14%), sunflower oil, saffron oil, salt	135	166	184	191
Rice	Conventional	Auchan		rice (14%), sunflower oil, salt	137	145	200	
Rice	Conventional	Alpro		rice (not grown in the European Union) (12.5%), sunflower oil, salt, gellan gum, vitamins (B_12_, D_2_)	66	97	109	
Rice	Conventional	Alpro	dolce	rice (16%), sunflower oil, rapeseed lecithin, salt, gellan gum, vitamins (B_2_, B_12_, D)	37	39	64	
Rice	Conventional	Plant Pro		rice (15%), sunflower oil, salt	80	99	117	
Rice	Organic	DMbio	natural	rice (13%), sunflower oil, salt	32	47	49	
Rice	Organic	Happy		rice (12.1%), sunflower oil, calcium carbonate, gellan gum, salt	179	180	206	
Rice	Organic	Isola	with added calcium	rice (17%), sunflower oil, seaweed, salt	176	188	193	
Rice	Organic	Riso Scotti	with added calcium	rice (17%), sunflower oil, seaweed, salt	88	104	112	
Rice	Organic	The Bridge	gluten-free, with added calcium	Italian rice (17%), sunflower oil, saffron oil, seaweed, sea salt	83	98	105	
Rice	Organic	The Bridge	gluten-free; natural	Italian rice (17%), sunflower oil, saffron oil, sea salt	69	77	100	120
Rice	Organic	My Bio		rice (17%), sunflower oil, salt	159	165	171	
Soy	Conventional	Alpro	low sugar	soybean (8%), sugar, gellan gum, sea salt, vitamins (B_2_, B_12_, D_2_)	1	7	33	
Soy	Conventional	Alpro	sugar-free	shelled soybean (8.7%), calcium carbonate, salt, gellan gum, vitamins (B_2_, B_12_, D_2_)	175	186	195	
Soy	Organic	DMbio	with added calcium	soybean (7%), cane sugar, seaweed, salt	34	60	62	
Soy	Organic	DMbio	natural	soybean (8%)	78	94	116	
Soy	Organic	Happy	original recipe	soybean (6.9%), sugar, calcium carbonate, gellan gum, disodium phosphate, vitamins (B_2_, D, B_12_)	197			
Spelt	Organic	DMbio	natural	spelt (11%), sunflower oil, salt	87	108	122	

**Table 2:** The BreathSpec GC-IMS carrier gas profile. GC-IMS, gas chromatography with ion mobility spectrometry; time, the length of the step (minutes:seconds); | indicates open.

Time	Carrier Gas Flow Rate [mL/min]
00:00.000	|
00:00.500	5.0
00:09.500	5.0
02:00.000	2.0
10:00.000	2.0
25:00.020	60.0

**Table 3:** Summary of product characteristics evaluated by PCA. We listed the main ingredients that overlapping groups shared. The ratios of overlapping groups are also shown. When we compared the GC-IMS and the electronic nose, we marked the better result with green, the same performance with black, and the worse result with red. A higher number of red results were seen in GC-IMS results. PCA, principal component analysis; GC-IMS, gas chromatography with ion mobility spectrometry; conv., conventional; org., organic.

GC-IMS					Electronic Nose				
Brand	Type	Rate of Overlapping	Main Overlapping Ingredients	Notable Findings	Brand	Type	Rate of Overlapping	Main Overlapping Ingredients	Notable Findings
Alpro	Almond	1/3	- Carob flour - Gellan gum - -Vitamin E - Sunflower-lecithin - Vitamin B_2_ - Vitamin B_12_ - Vitamin D_2_ - Vitamin E	Complete separation of soy samples	Alpro	Almond	0/3	- Gellan gum - Salt - Vitamin B_12_ - Vitamin D_2_	Complete separation of almond and cashew samples
	Cashew	1/3				Cashew	0/3		
	Rice	2/3				Rice	1/3		
	Soy (s.f.)	0/3				Soy (s.f.)	1/3		
Barista	Almond	2/3	- Salt - Sunflower oil	Coconut and oat 2 samples are difficult to distinguish	Barista	Almond	2/3	- Salt - Sunflower oil	Complete separation of coconut samples
	Coconut	3/3				Coconut	0/3		
	Oat 1	2/3				Oat 1	2/3		
	Oat 2	3/3				Oat 2	2/3		
DMbio	Almond	3/5	- Salt - Sunflower oil	Oat, soy, and spelt samples are difficult to distinguish	DMbio	Almond	0/5	- Salt - Sunflower oil	- Complete separation of almond samples - Oat, soy and spelt samples are difficult to distinguish
	Coconut	4/5				Coconut	3/5		
	Oat	5/5				Oat	4/5		
	Rice	4/5				Rice	3/5		
	Soy	5/5				Soy	4/5		
	Spelt	5/5				Spelt	4/5		
Almond	Barista	2/3	- Almond - Calcium-carbonate - Carob flour - Gellan gum - Guar gum - Salt - Sugar - Sunflower-lecithin - Vitamin B_12_ - Vitamin D - Vitamin E	Conventional and organic almond samples are difficult to distinguish	Almond	Barista	1/3	- Almond - Salt	Complete separation of conventional and roasted conventional samples
	Conventional	3/3				Conventional	0/3		
	Organic	3/3				Organic	1/3		
	Roasted (conv.)	2/3				Roasted (conv.)	0/3		
Coconut	Adez	3/4	- Coconut - Gellan gum - Guar gum - Salt - Rice - -Vitamin B_12_ - Vitamin D_2_	Happy, Joya, and Naturaktiv samples are difficult to distinguish	Coconut	Adez	3/4	- Coconut - Gellan gum - Guar gum - Salt - Rice - Vitamin B_12_ - Vitamin D_2_	Joya and Naturaktiv samples are difficult to distinguish
	Happy	4/4				Happy	3/4		
	Joya	4/4				Joya	4/4		
	Koko	3/4				Koko	2/4		
	Naturaktiv	4/4				Naturaktiv	4/4		
Rice (conv.)	Alpro	2/3	- Gellan gum - Rice - Salt - Sunflower oil-Vitamin B_12_	Complete separation of Happy samples	Rice (conv.)	Alpro	0/3	- Gellan gum - Rice - Salt - Sunflower oil	- Complete separation of Alpro samples - Alpro (sweet), Happy and PlantPro samples are difficult to distinguish
	Alpro (sweet)	1/3				Alpro (sweet)	2/3		
	Happy	0/3				Happy	2/3		
	PlantPro	1/3				PlantPro	2/3		
Rice (org.)	Auchan	4/5	- Rice - Salt - Seaweed - Sunflower oil	DMbio, Isola, Riso Scotti, and The Bridge samples are difficult to distinguish	Rice (org.)	Auchan	3/5	- Rice - Salt - Seaweed - Sunflower oil	Isola, MyBio and The Bridge samples are difficult to distinguish
	DMbio	5/5				DMbio	4/5		
	Isola	5/5				Isola	5/5		
	MyBio	4/5				MyBio	5/5		
	Riso Scotti	5/5				Riso Scotti	4/5		
	The Bridge	5/5				The Bridge	5/5		

**Table 4:** The percentages of correct classification by LDA before and after cross-validation.

Examined Group	GC-IMS		Electronic Nose	
	Original Grouped cases Correctly Classified	Cross-Validated Grouped Cases Correctly Classified	Original Grouped Cases Correctly Classified	Cross-Validated Grouped Cases Correctly Classified
Alpro	100.0%	15.4%	100.0%	100.0%
Barista	100.0%	92.3%	100.0%	100.0%
DMbio	100.0%	89.5%	100.0%	100.0%
Almond	100.0%	95.0%	100.0%	100.0%
Coconut	100.0%	100.0%	100.0%	96.2%
Rice (convent.)	100.0%	91.7%	100.0%	100.0%
Rice (organic)	100.0%	90.9%	100.0%	100.0%

LDA, linear discrimination analysis; GC-IMS, gas chromatography with ion mobility spectroscopy; convent., conventional.

## Data Availability

The original contributions presented in the study are included in the article/supplementary material, further inquiries can be directed to the corresponding author.

## Supplementary Materials

The following supporting information can be downloaded at: www.mdpi.com/10.3390/foods13244086/s1, Table S1. Classification results of the LDA analysis of plant-based drinks by the brand “Alpro” analysed with GC-IMS. Table S2. Classification results of the LDA analysis of plant-based drinks by the type “Barista” analysed with GC-IMS. Table S3. Classification results of the LDA analysis of plant-based drinks by the brand “DMbio” analysed with GC-IMS. Table S4. Classification results of the LDA analysis of plant-based almond drinks analysed with GC-IMS. Table S5. Classification results of the LDA analysis of plant-based coconut drinks analysed with GC-IMS. Table S6. Classification results of the LDA analysis of conventional plant-based rice drinks analysed with GC-IMS. Table S7. Classification results of the LDA analysis of organic plant-based rice drinks analysed with GC-IMS. Table S8. Classification results of the LDA analysis of plant-based drinks by the brand “Alpro” analysed with Electronic nose. Table S9. Classification results of the LDA analysis of plant-based drinks by the type “Barista” analysed with Electronic nose. Table S10. Classification results of the LDA analysis of plant-based drinks by the brand “DMbio” analysed with Electronic nose. Table S11. Classification results of the LDA analysis of plant-based almond drinks analysed with Electronic nose. Table S12. Classification results of the LDA analysis of plant-based coconut drinks analysed with Electronic nose. Table S13. Classification results of the LDA analysis of conventional plant-based rice drinks analysed with Electronic nose. Table S14. Classification results of the LDA analysis of organic plant-based rice drinks analysed with Electronic nose.
