# Supplementary material for: Classification of Plant-Based Drinks Based on Volatile Compounds
Source: Foods. 2024 Dec 17;13(24):4086. doi: 10.3390/foods13244086 (PMC11675735; doi:10.3390/foods13244086)
Supplement: Supplementary file 1 [file foods-13-04086-s001.zip › foods-3287970-supplementary.pdf]

foods MDPI

Supplementary material

Classification of plant-based drinks based on volatile compounds

Zsigmond Papp [1], Laura Nemeth [1,2], Sandrine Nzetchouang Siyapndjeu [1,2,3], Anita Bufa [4], Tamás Marosvölgyi [4] and Zoltán Gyöngyi [1,*]

[1] Department of Public Health Medicine, Medical School, University of Pécs, Szigeti út, 12, 7624 Pécs, Hungary; papp.zsigmond@pte.hu; laura.nemeth@pte.hu, sandrine.nzetchouangsiyapndjeu@aphp.fr
[2] Faculty of Health Sciences, University of Pécs, Vörösmarty M. u. 4, 7621 Pécs, Hungary; laura.nemeth@pte.hu, sandrine.nzetchouangsiyapndjeu@aphp.fr
[3] Unité de Recherche Clinique, GHU Paris Centre, Université Paris Cité, 89 rue d'Assas, 75006 Paris, France, France; sandrine.nzetchouangsiyapndjeu@aphp.fr
[4] Institute of Bioanalysis, Medical School, University of Pécs, Szigeti út, 12, 7624 Pécs, Hungary; anita.bufa@aok.pte.hu; marosvolgyi.tamas@pte.hu
* Correspondence: zoltan.gyongyi@aok.pte.hu

Table S1. Classification results of the LDA analysis of plant-based drinks by the brand „Alpro" analysed with GC-IMS.

Classification Results[a,c]

			Predicted Group Membership				
		VAR00003	1.00	2.00	3.00	4.00	Total
Original	Count	1.00	4	0	0	0	4
		2.00	0	4	0	0	4
		3.00	0	0	3	0	3
		4.00	0	0	0	2	2
	%	1.00	100.0	.0	.0	.0	100.0
		2.00	.0	100.0	.0	.0	100.0
		3.00	.0	.0	100.0	.0	100.0
		4.00	.0	.0	.0	100.0	100.0
Cross-validated[b]	Count	1.00	0	0	4	0	4
		2.00	0	1	3	0	4
		3.00	0	0	0	3	3
		4.00	1	0	0	1	2
	%	1.00	.0	.0	100.0	.0	100.0
		2.00	.0	25.0	75.0	.0	100.0
		3.00	.0	.0	.0	100.0	100.0
		4.00	50.0	.0	.0	50.0	100.0

a. 100.0% of original grouped cases are correctly classified.

b. Cross-validation is done only for the cases in the analysis. In cross-validation, each case is classified by the functions derived from all cases other than that case.

c. 15.4% of cross-validated grouped cases are correctly classified.

Table S2. Classification results of the LDA analysis of plant-based drinks by the type „Barista" analysed with GC-IMS.

Classification Results[a,c]

		VAR00003	Predicted Group Membership 1.00	2.00	3.00	4.00	Total
Original	Count	1.00	4	0	0	0	4
		2.00	0	2	0	0	2
		3.00	0	0	3	0	3
		4.00	0	0	0	4	4
	%	1.00	100.0	.0	.0	.0	100.0
		2.00	.0	100.0	.0	.0	100.0
		3.00	.0	.0	100.0	.0	100.0
		4.00	.0	.0	.0	100.0	100.0
Cross-validated[b]	Count	1.00	4	0	0	0	4
		2.00	0	2	0	0	2
		3.00	0	0	3	0	3
		4.00	1	0	0	3	4
	%	1.00	100.0	.0	.0	.0	100.0
		2.00	.0	100.0	.0	.0	100.0
		3.00	.0	.0	100.0	.0	100.0
		4.00	25.0	.0	.0	75.0	100.0

a. 100.0% of original grouped cases are correctly classified.

b. Cross-validation is done only for the cases in the analysis. In cross-validation, each case is classified by the functions derived from all cases other than that case.

c. 92.3% of cross-validated grouped cases are correctly classified.

Table S3. Classification results of the LDA analysis of plant-based drinks by the brand „DMbio" analysed with GC-IMS.

Classification Results[a,c]

		VAR00003	Predicted Group Membership 1.00	2.00	3.00	4.00	5.00	6.00	Total
Original	Count	1.00	3	0	0	0	0	0	3
		2.00	0	2	0	0	0	0	2
		3.00	0	0	3	0	0	0	3
		4.00	0	0	0	3	0	0	3
		5.00	0	0	0	0	3	0	3
		6.00	0	0	0	0	0	5	5
	%	1.00	100.0	.0	.0	.0	.0	.0	100.0
		2.00	.0	100.0	.0	.0	.0	.0	100.0

		3.00	.0	.0	100.0	.0	.0	.0	100.0
		4.00	.0	.0	.0	100.0	.0	.0	100.0
		5.00	.0	.0	.0	.0	100.0	.0	100.0
		6.00	.0	.0	.0	.0	.0	100.0	100.0
Cross-validated[b]	Count	1.00	3	0	0	0	0	0	3
		2.00	0	2	0	0	0	0	2
		3.00	0	0	2	1	0	0	3
		4.00	0	0	0	3	0	0	3
		5.00	0	0	0	0	3	0	3
		6.00	0	0	1	0	0	4	5
	%	1.00	100.0	.0	.0	.0	.0	.0	100.0
		2.00	.0	100.0	.0	.0	.0	.0	100.0
		3.00	.0	.0	66.7	33.3	.0	.0	100.0
		4.00	.0	.0	.0	100.0	.0	.0	100.0
		5.00	.0	.0	.0	.0	100.0	.0	100.0
		6.00	.0	.0	20.0	.0	.0	80.0	100.0

a. 100.0% of original grouped cases are correctly classified.

b. Cross-validation is done only for the cases in the analysis. In cross-validation, each case is classified by the functions derived from all cases other than that case.

c. 89.5% of cross-validated grouped cases are correctly classified.

Table S4. Classification results of the LDA analysis of plant-based almond drinks analysed with GC-IMS.

Classification Results[a,c]

			Predicted Group Membership				
		VAR00003	1.00	2.00	3.00	4.00	Total
Original	Count	1.00	4	0	0	0	4
		2.00	0	4	0	0	4
		3.00	0	0	6	0	6
		4.00	0	0	0	6	6
	%	1.00	100.0	.0	.0	.0	100.0
		2.00	.0	100.0	.0	.0	100.0
		3.00	.0	.0	100.0	.0	100.0
		4.00	.0	.0	.0	100.0	100.0
Cross-validated[b]	Count	1.00	4	0	0	0	4
		2.00	0	4	0	0	4
		3.00	1	0	5	0	6
		4.00	0	0	0	6	6
	%	1.00	100.0	.0	.0	.0	100.0
		2.00	.0	100.0	.0	.0	100.0
		3.00	16.7	.0	83.3	.0	100.0

	4.00	.0	.0	.0	100.0	100.0

a. 100.0% of original grouped cases are correctly classified.

b. Cross-validation is done only for the cases in the analysis. In cross-validation, each case is classified by the functions derived from all cases other than that case.

c. 95.0% of cross-validated grouped cases are correctly classified.

Table S5. Classification results of the LDA analysis of plant-based coconut drinks analysed with GC-IMS.

Classification Results[a,c]

			Predicted Group Membership					
		VAR00003	1.00	2.00	3.00	4.00	5.00	Total
Original	Count	1.00	4	0	0	0	0	4
		2.00	0	3	0	0	0	3
		3.00	0	0	3	0	0	3
		4.00	0	0	0	3	0	3
		5.00	0	0	0	0	3	3
	%	1.00	100.0	.0	.0	.0	.0	100.0
		2.00	.0	100.0	.0	.0	.0	100.0
		3.00	.0	.0	100.0	.0	.0	100.0
		4.00	.0	.0	.0	100.0	.0	100.0
		5.00	.0	.0	.0	.0	100.0	100.0
Cross-validated[b]	Count	1.00	4	0	0	0	0	4
		2.00	0	3	0	0	0	3
		3.00	0	0	3	0	0	3
		4.00	0	0	0	3	0	3
		5.00	0	0	0	0	3	3
	%	1.00	100.0	.0	.0	.0	.0	100.0
		2.00	.0	100.0	.0	.0	.0	100.0
		3.00	.0	.0	100.0	.0	.0	100.0
		4.00	.0	.0	.0	100.0	.0	100.0
		5.00	.0	.0	.0	.0	100.0	100.0

a. 100.0% of original grouped cases are correctly classified.

b. Cross-validation is done only for the cases in the analysis. In cross-validation, each case is classified by the functions derived from all cases other than that case.

c. 100.0% of cross-validated grouped cases are correctly classified.

Table S6. Classification results of the LDA analysis of conventional plant-based rice drinks analysed with GC-IMS.

Classification Results[a,c]

			Predicted Group Membership				
		VAR00003	1.00	2.00	3.00	4.00	Total

Original	Count	1.00	4	0	0	0	4
		2.00	0	3	0	0	3
		3.00	0	0	2	0	2
		4.00	0	0	0	3	3
	%	1.00	100.0	.0	.0	.0	100.0
		2.00	.0	100.0	.0	.0	100.0
		3.00	.0	.0	100.0	.0	100.0
		4.00	.0	.0	.0	100.0	100.0
Cross-validated[b]	Count	1.00	3	1	0	0	4
		2.00	0	3	0	0	3
		3.00	0	0	2	0	2
		4.00	0	0	0	3	3
	%	1.00	75.0	25.0	.0	.0	100.0
		2.00	.0	100.0	.0	.0	100.0
		3.00	.0	.0	100.0	.0	100.0
		4.00	.0	.0	.0	100.0	100.0

a. 100.0% of original grouped cases are correctly classified.

b. Cross-validation is done only for the cases in the analysis. In cross-validation, each case is classified by the functions derived from all cases other than that case.

c. 91.7% of cross-validated grouped cases are correctly classified.

Table S7. Classification results of the LDA analysis of organic plant-based rice drinks analysed with GC-IMS.

Classification Results[a,c]

			Predicted Group Membership						
		VAR00003	1.00	2.00	3.00	4.00	5.00	6.00	Total
Original	Count	1.00	2	0	0	0	0	0	2
		2.00	0	3	0	0	0	0	3
		3.00	0	0	5	0	0	0	5
		4.00	0	0	0	3	0	0	3
		5.00	0	0	0	0	6	0	6
		6.00	0	0	0	0	0	3	3
	%	1.00	100.0	.0	.0	.0	.0	.0	100.0
		2.00	.0	100.0	.0	.0	.0	.0	100.0
		3.00	.0	.0	100.0	.0	.0	.0	100.0
		4.00	.0	.0	.0	100.0	.0	.0	100.0
		5.00	.0	.0	.0	.0	100.0	.0	100.0
		6.00	.0	.0	.0	.0	.0	100.0	100.0
Cross-validated[b]	Count	1.00	2	0	0	0	0	0	2
		2.00	0	3	0	0	0	0	3

		3.00	0	0	4	1	0	0	5
		4.00	0	0	0	3	0	0	3
		5.00	0	0	1	0	5	0	6
		6.00	0	0	0	0	0	3	3
	%	1.00	100.0	.0	.0	.0	.0	.0	100.0
		2.00	.0	100.0	.0	.0	.0	.0	100.0
		3.00	.0	.0	80.0	20.0	.0	.0	100.0
		4.00	.0	.0	.0	100.0	.0	.0	100.0
		5.00	.0	.0	16.7	.0	83.3	.0	100.0
		6.00	.0	.0	.0	.0	.0	100.0	100.0

a. 100.0% of original grouped cases are correctly classified.

b. Cross-validation is done only for the cases in the analysis. In cross-validation, each case is classified by the functions derived from all cases other than that case.

c. 90.9% of cross-validated grouped cases are correctly classified.

Table S8. Classification results of the LDA analysis of plant-based drinks by the brand „Alpro" analysed with Electronic nose.

Classification Results[a,c]

			Predicted Group Membership				
		VAR00003	1.00	2.00	3.00	4.00	Total
Original	Count	1.00	20	0	0	0	20
		2.00	0	15	0	0	15
		3.00	0	0	15	0	15
		4.00	0	0	0	10	10
	%	1.00	100.0	.0	.0	.0	100.0
		2.00	.0	100.0	.0	.0	100.0
		3.00	.0	.0	100.0	.0	100.0
		4.00	.0	.0	.0	100.0	100.0
Cross-validated[b]	Count	1.00	20	0	0	0	20
		2.00	0	15	0	0	15
		3.00	0	0	15	0	15
		4.00	0	0	0	10	10
	%	1.00	100.0	.0	.0	.0	100.0
		2.00	.0	100.0	.0	.0	100.0
		3.00	.0	.0	100.0	.0	100.0
		4.00	.0	.0	.0	100.0	100.0

a. 100.0% of original grouped cases are correctly classified.

b. Cross-validation is done only for the cases in the analysis. In cross-validation, each case is classified by the functions derived from all cases other than that case.

c. 100.0% of cross-validated grouped cases are correctly classified.

Table S9. Classification results of the LDA analysis of plant-based drinks by the type „Barista" analysed with Electronic nose.

Classification Results[a,c]

		VAR00003	Predicted Group Membership 1.00	2.00	3.00	4.00	Total
Original	Count	1.00	8	0	0	0	8
		2.00	0	10	0	0	10
		3.00	0	0	15	0	15
		4.00	0	0	0	19	19
	%	1.00	100.0	.0	.0	.0	100.0
		2.00	.0	100.0	.0	.0	100.0
		3.00	.0	.0	100.0	.0	100.0
		4.00	.0	.0	.0	100.0	100.0
Cross-validated[b]	Count	1.00	8	0	0	0	8
		2.00	0	10	0	0	10
		3.00	0	0	15	0	15
		4.00	0	0	0	19	19
	%	1.00	100.0	.0	.0	.0	100.0
		2.00	.0	100.0	.0	.0	100.0
		3.00	.0	.0	100.0	.0	100.0
		4.00	.0	.0	.0	100.0	100.0

a. 100.0% of original grouped cases are correctly classified.

b. Cross-validation is done only for the cases in the analysis. In cross-validation, each case is classified by the functions derived from all cases other than that case.

c. 100.0% of cross-validated grouped cases are correctly classified.

Table S10. Classification results of the LDA analysis of plant-based drinks by the brand „DMbio" analysed with Electronic nose.

Classification Results[a,c]

		VAR00003	Predicted Group Membership 1.00	2.00	3.00	4.00	5.00	6.00	Total
Original	Count	1.00	14	0	0	0	0	0	14
		2.00	0	10	0	0	0	0	10
		3.00	0	0	15	0	0	0	15
		4.00	0	0	0	15	0	0	15
		5.00	0	0	0	0	15	0	15
		6.00	0	0	0	0	0	25	25

	%	1.00	100.0	.0	.0	.0	.0	.0	100.0
		2.00	.0	100.0	.0	.0	.0	.0	100.0
		3.00	.0	.0	100.0	.0	.0	.0	100.0
		4.00	.0	.0	.0	100.0	.0	.0	100.0
		5.00	.0	.0	.0	.0	100.0	.0	100.0
		6.00	.0	.0	.0	.0	.0	100.0	100.0
Cross-validated[b]	Count	1.00	14	0	0	0	0	0	14
		2.00	0	10	0	0	0	0	10
		3.00	0	0	15	0	0	0	15
		4.00	0	0	0	15	0	0	15
		5.00	0	0	0	0	15	0	15
		6.00	0	0	0	0	0	25	25
	%	1.00	100.0	.0	.0	.0	.0	.0	100.0
		2.00	.0	100.0	.0	.0	.0	.0	100.0
		3.00	.0	.0	100.0	.0	.0	.0	100.0
		4.00	.0	.0	.0	100.0	.0	.0	100.0
		5.00	.0	.0	.0	.0	100.0	.0	100.0
		6.00	.0	.0	.0	.0	.0	100.0	100.0

a. 100.0% of original grouped cases are correctly classified.

b. Cross-validation is done only for the cases in the analysis. In cross-validation, each case is classified by the functions derived from all cases other than that case.

c. 100.0% of cross-validated grouped cases are correctly classified.

Table S11. Classification results of the LDA analysis of plant-based almond drinks analysed with Electronic nose.

Classification Results[a,c]

			Predicted Group Membership				
		VAR00003	1.00	2.00	3.00	4.00	Total
Original	Count	1.00	20	0	0	0	20
		2.00	0	18	0	0	18
		3.00	0	0	30	0	30
		4.00	0	0	0	27	27
	%	1.00	100.0	.0	.0	.0	100.0
		2.00	.0	100.0	.0	.0	100.0
		3.00	.0	.0	100.0	.0	100.0
		4.00	.0	.0	.0	100.0	100.0
Cross-validated[b]	Count	1.00	20	0	0	0	20
		2.00	0	18	0	0	18
		3.00	0	0	30	0	30
		4.00	0	0	0	27	27

	%	1.00	100.0	.0	.0	.0	100.0
		2.00	.0	100.0	.0	.0	100.0
		3.00	.0	.0	100.0	.0	100.0
		4.00	.0	.0	.0	100.0	100.0

a. 100.0% of original grouped cases are correctly classified.

b. Cross-validation is done only for the cases in the analysis. In cross-validation, each case is classified by the functions derived from all cases other than that case.

c. 100.0% of cross-validated grouped cases are correctly classified.

Table S12. Classification results of the LDA analysis of plant-based coconut drinks analysed with Electronic nose.

Classification Results[a,c]

			Predicted Group Membership					
		VAR00003	1.00	2.00	3.00	4.00	5.00	Total
Original	Count	1.00	20	0	0	0	0	20
		2.00	0	15	0	0	0	15
		3.00	0	0	14	0	0	14
		4.00	0	0	0	14	0	14
		5.00	0	0	0	0	15	15
	%	1.00	100.0	.0	.0	.0	.0	100.0
		2.00	.0	100.0	.0	.0	.0	100.0
		3.00	.0	.0	100.0	.0	.0	100.0
		4.00	.0	.0	.0	100.0	.0	100.0
		5.00	.0	.0	.0	.0	100.0	100.0
Cross-validated[b]	Count	1.00	20	0	0	0	0	20
		2.00	0	15	0	0	0	15
		3.00	0	1	13	0	0	14
		4.00	0	0	1	12	1	14
		5.00	0	0	0	0	15	15
	%	1.00	100.0	.0	.0	.0	.0	100.0
		2.00	.0	100.0	.0	.0	.0	100.0
		3.00	.0	7.1	92.9	.0	.0	100.0
		4.00	.0	.0	7.1	85.7	7.1	100.0
		5.00	.0	.0	.0	.0	100.0	100.0

a. 100.0% of original grouped cases are correctly classified.

b. Cross-validation is done only for the cases in the analysis. In cross-validation, each case is classified by the functions derived from all cases other than that case.

c. 96.2% of cross-validated grouped cases are correctly classified.

Table S13. Classification results of the LDA analysis of conventional plant-based rice drinks analysed with Electronic nose.

Classification Results[a,c]

			Predicted Group Membership				
		VAR00003	1.00	2.00	3.00	4.00	Total
Original	Count	1.00	15	0	0	0	15
		2.00	0	15	0	0	15
		3.00	0	0	10	0	10
		4.00	0	0	0	15	15
	%	1.00	100.0	.0	.0	.0	100.0
		2.00	.0	100.0	.0	.0	100.0
		3.00	.0	.0	100.0	.0	100.0
		4.00	.0	.0	.0	100.0	100.0
Cross-validated[b]	Count	1.00	15	0	0	0	15
		2.00	0	15	0	0	15
		3.00	0	0	10	0	10
		4.00	0	0	0	15	15
	%	1.00	100.0	.0	.0	.0	100.0
		2.00	.0	100.0	.0	.0	100.0
		3.00	.0	.0	100.0	.0	100.0
		4.00	.0	.0	.0	100.0	100.0

a. 100.0% of original grouped cases correctly classified.

b. Cross-validation is done only for the cases in the analysis. In cross-validation, each case is classified by the functions derived from all cases other than that case.

c. 100.0% of cross-validated grouped cases correctly classified.

Table S14. Classification results of the LDA analysis of organic plant-based rice drinks analysed with Electronic nose.

Classification Results[a,c]

		VAR00	Predicted Group Membership						
		003	1.00	2.00	3.00	4.00	5.00	6.00	Total
Original	Count	1.00	10	0	0	0	0	0	10
		2.00	0	15	0	0	0	0	15
		3.00	0	0	25	0	0	0	25
		4.00	0	0	0	15	0	0	15
		5.00	0	0	0	0	28	0	28
		6.00	0	0	0	0	0	14	14
	%	1.00	100.0	.0	.0	.0	.0	.0	100.0
		2.00	.0	100.0	.0	.0	.0	.0	100.0
		3.00	.0	.0	100.0	.0	.0	.0	100.0
		4.00	.0	.0	.0	100.0	.0	.0	100.0

		5.00	.0	.0	.0	.0	100.0	.0	100.0
		6.00	.0	.0	.0	.0	.0	100.0	100.0
Cross-validated[b]	Count	1.00	10	0	0	0	0	0	10
		2.00	0	15	0	0	0	0	15
		3.00	0	0	25	0	0	0	25
		4.00	0	0	0	15	0	0	15
		5.00	0	0	0	0	28	0	28
		6.00	0	0	0	0	0	14	14
	%	1.00	100.0	.0	.0	.0	.0	.0	100.0
		2.00	.0	100.0	.0	.0	.0	.0	100.0
		3.00	.0	.0	100.0	.0	.0	.0	100.0
		4.00	.0	.0	.0	100.0	.0	.0	100.0
		5.00	.0	.0	.0	.0	100.0	.0	100.0
		6.00	.0	.0	.0	.0	.0	100.0	100.0

a. 100.0% of original grouped cases are correctly classified.

b. Cross-validation is done only for the cases in the analysis. In cross-validation, each case is classified by the functions derived from all cases other than that case.

c. 100.0% of cross-validated grouped cases are correctly classified.
